# LytB1 and LytB2 of *Mycobacterium tuberculosis* Are Not Genetically Redundant

**DOI:** 10.1371/journal.pone.0135638

**Published:** 2015-08-26

**Authors:** Amanda Claire Brown, Rachel Kokoczka, Tanya Parish

**Affiliations:** 1 Queen Mary University of London, London, E1 2AD, United Kingdom; 2 Barts & the London School of Medicine and Dentistry, London, E1 2AD, United Kingdom; 3 TB Discovery Research, Infectious Disease Research Institute, Seattle, WA, 98102, United States of America; Centre National de la Recherche Scientifique—Université de Toulouse, FRANCE

## Abstract

*Mycobacterium tuberculosis* synthesises isoprenoid precursors via the MEP/DOXP pathway and at least five enzymes in the pathway (Dxs1, Dxr/IspC, IspD, IspF, and GcpE/IspG) are required for growth in vitro. We investigated the role of LytB (IspH) in *M*. *tuberculosis*; *M*. *tuberculosis* is unusual in that it has two homologs–LytB1 and LytB2. We were unable to delete the *lytB2* gene unless we provided an additional copy elsewhere, demonstrating that this is the essential homolog. We expressed *lytB1* from the *lytB2* promoter and confirmed that this could not complement for loss of function of *lytB2*, despite LytB1 possessing all the previously described conserved critical residues. Interestingly the sole LytB homolog of *Mycobacterium smegmatis* was able to compensate for loss of LytB2 in *M*. *tuberculosis*. We tested translational fusions of LytB1 and LytB2 for functionality in *M*. *tuberculosis*, but only a fusion with 90% N-terminal LytB2 and 10% C-terminal LytB1 was functional. In order to identify the key difference between the two proteins, site directed mutagenesis was used to change LytB2 residues into their counterparts in LytB1. None of these amino acid substitutions was essential for function and all *lytB2* mutant alleles were functional. In contrast, mutation of the key residues for [Fe4S4] cluster formation, as well as a catalytic residue in LytB1 did not result in functional complementation. Thus, although LytB1 and LytB2 are not genetically redundant, this is not dependent on small amino acid changes, but is likely to be a result of major overall structural differences.

## Introduction

*Mycobacterium tuberculosis*, the causative agent of human tuberculosis, poses an increasing threat to human health [1]. The escalating incidence of drug resistant strains provides a new impetus to understand this complex pathogen and to identify metabolic targets for novel therapeutics. As a first step towards this aim, we have been directing our efforts to identifying metabolic pathways essential for bacterial viability [2–4].

Several of the current antibiotics active against *M*. *tuberculosis* target cell wall biosynthesis, in particular the synthesis of mycolic acids, inhibited by isoniazid and ethionamide, or arabinogalactan and lipoarabinomannan, inhibited by ethambutol [5,6]. Isoprenoid biosynthesis is a key synthetic pathway required for the generation of many cellular components, including cell wall components [7]. *M*. *tuberculosis* synthesises the isoprenoid precursor, isopentenyl diphosphate (IPP), via the non-mevalonate or 1-deoxy-D-xylulose 5-phosphate (MEP/DOXP) pathway, in contrast to the human mevalonate pathway [2,8]. Therefore the bacterial enzymes can be specifically targeted without interfering with eukaryotic isoprenoid biosynthesis, making this an attractive pathway in the search for novel drugs.

The MEP/DOXP pathway has been characterised in several bacterial species; all of the genes required can be identified by homology in the *M*. *tuberculosis* genome and the proposed pathway can be reconstructed (S1 Fig) [2,3,9]. Recombinant proteins have been produced and enzymatic activities have been confirmed for Dxs1 [10], Dxr/IspC [11,12], IspD [13], IspE [13] and IspF [4]. In addition, we have previously demonstrated that Dxs1, Dxr/IspC, IspD, IspF, and GcpE/IspG are all essential for the *in vitro* growth of *M*. *tuberculosis* [2–4,13].

LytB (IspH) is a 4-hydroxy-3-methylbut-2-enyl diphosphate reductase (HDR), it acts as the terminal step of the MEP/DOXP pathway catalyzing the conversion of (*E*)-4-hydroxy-3-methyl-but-2-enyl pyrophosphate (HMB-PP) into IPP and dimethylallyl pyrophosphate (DMAPP). The enzyme appears to be responsible for the parallel production of both IPP and DMAPP, formed in a ~5:1 ratio, although the exact mechanism of this is not currently fully understood [14]. The LytB structure has been determined in several species and the enzyme contains an iron-sulphur [Fe_4_S_4_] cluster [14–22]. A number of highly conserved critical residues have been identified in the HDRs from *Escherichia coli*, *Aquifex aeolicus*, *Arabidopsis thalina* and *Plasmodium falciparum* [14,21,23–25]. These include residues required for Fe-S cluster formation (*E*. *coli* numbering Cys-12, Cys-96, Cys-197) [14–17,24,25], for the delivery of H^+^ to the active site (Glu-126) [25,26], and for ligand docking of HMB-PP into the central cavity of LytB (His-41, His-124, Thr-167, Ser-225, Asn-227) [17,20,22,24,25].

**Fig 1:**
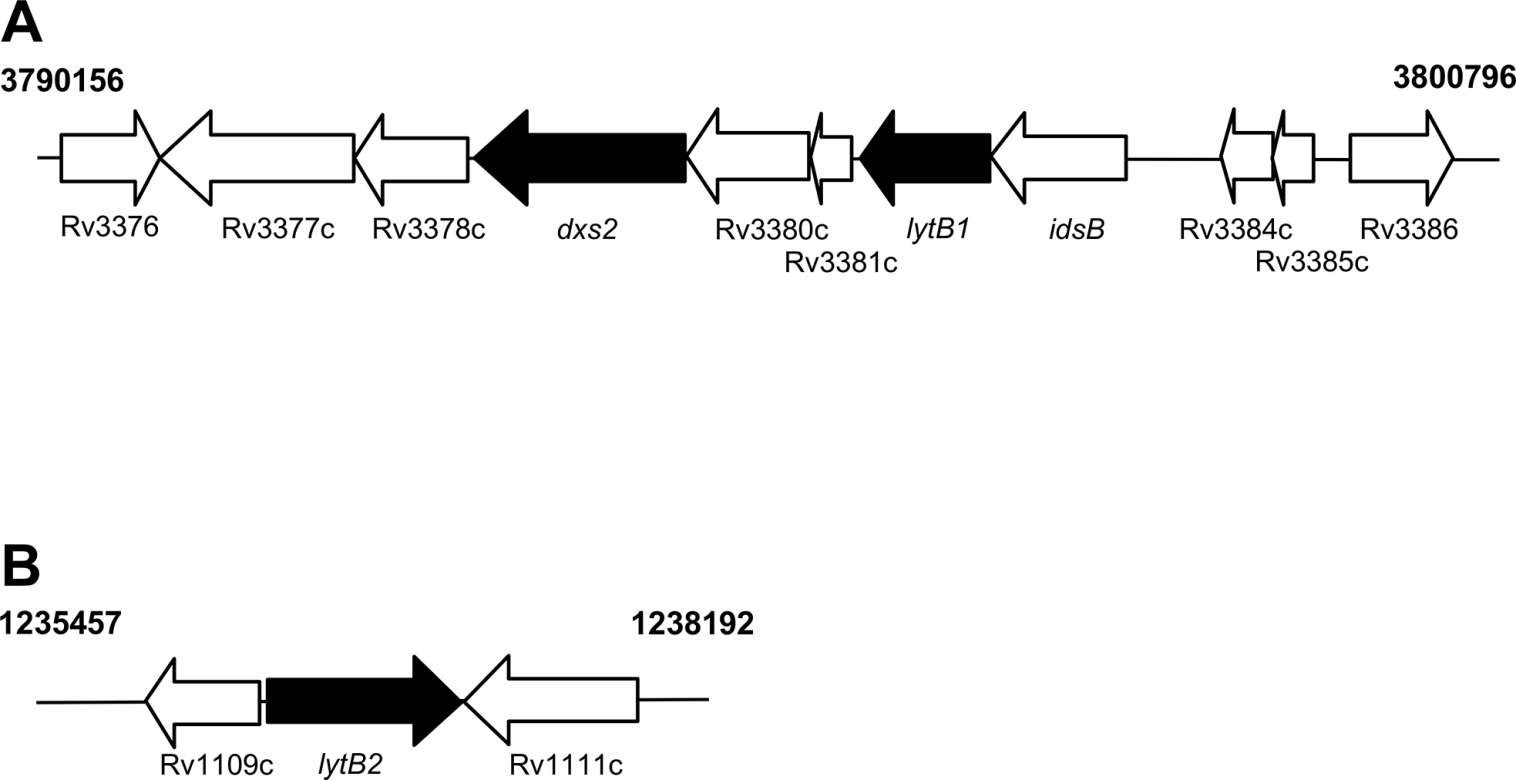
Genomic organisation of *lytB*. The chromosomal arrangement is indicated for *lytB1* (A) and *lytB2* (B) in *M*. *tuberculosis*. DOXP pathway genes are in black. The genetic co-ordinates for the H37Rv strain of *M*. *tuberculosis* are given. Typically in mycobacteria *lytB1* is expressed in an operon with *dxs1*, whereas *lytB2* is expressed alone.

The *M*. *tuberculosis* complex has two homologs of *lytB* (Rv3382c and Rv1110; Fig 1), whereas the related fast-growing non-pathogenic species *Mycobacterium smegmatis* has only one homolog (MSMEG_5224) as does *Mycobacterium leprae* (ML1938c) and *E*. *coli* (IspH). In *M*. *tuberculosis*, *lytB1* is located in an operon with *dxs2*, whereas *lytB2* is expressed independently (Fig 1) [9]. Mann *et al*., (2012) demonstrated that when expressed in *E*. *coli* both *lytb1* and *lytB2* produce enzymatically functional HDRs; although the LytB1 enzyme had lower activity compared with the LytB2 form [27]. Little is known of the expression or regulation of LytB1/2 in *M*. *tuberculosis*, although LytB expression is down-regulated in a cyctochrome *bc*_1_ mutant of *M*. *smegmatis* [28].

The MEP/DOXP pathway has been implicated in the pathogenic potential of mycobacterial species. The intermediate HMB-PP can activate gamma-delta T-cells [29,30]. Disruption of the non-essential *lytB1-dxs2* operon in *M*. *tuberculosis* abolishes the bacterium’s ability to prevent acidification of the phagosome and results in attenuated intracellular survival [31]. In addition, an *M*. *avium* subsp. *paratuberculosis gcpE* mutant has reduced ability to colonise tissue during infection of mice or calves [32,33], confirming that this pathway is required for virulence. However, in both cases, it is yet to be determined if this is a direct link between isoprenoid biosynthesis and the phenotypic consequences, since no measure of IPP or other intermediates was made.

We here describe the essentiality of LytB2 in *M*. *tuberculosis*, and confirm that LytB1 is not able functionally complement the loss of *lytB2*.

## Materials and Methods

### Culture of mycobacteria

*M*. *tuberculosis* H37Rv (ATCC 25618) was cultured in Middlebrook 7H9 liquid medium supplemented with 10% v/v OADC (oleic acid, bovine serum albumin, D-glucose, catalase; Becton Dickinson) and 0.05% w/v Tween 80 or on solid Middlebrook 7H10 agar supplemented with 10% v/v OADC at 37°C. X-gal (5-bromo-4-chloro-3-indolyl-ß-D-galactopyranoside) was used at 50 μg/mL, IPTG (isopropyl-beta-D-thiogalactopyranoside) at 0.5 mM, kanamycin at 20 μg/mL, hygromycin B at 100 μg/mL, gentamicin at 10 μg/mL, and sucrose at 2% w/v where required. *M*. *tuberculosis* growth curves were conducted in 16 mm glass tubes containing an 8 mm magnetic stirrer bar with stirring at 150 rpm.

### Determination of essentiality

A deletion delivery vector for *lytB2* (pLB2Δ) was constructed as follows: approximately 1 kb of the flanking regions surrounding the gene was amplified from *M*. *tuberculosis* genomic DNA using primer pairs LB2 Δ US F (PstI) 5’CCC CTG CAG CGG TCG ATG CGT GCC AGC AG 3’ and LB2 Δ US Rev (BamHI) 5’ CCC GGA TCC GGC TAC TGC ACC GTA TGG GG 3’; LB2 Δ DS F (BamHI) 5’-CCC GGA TCC CGC TGA GCA CAT CCG CTC AC 3’, and LB2 Δ DS Rev (HindIII) 5’ CCC AAG CTT GTG TGC CGT GGT GGG CTG CC 3’ (US/DS). Fragments were cloned into p2NIL using the PstI-HindIII restriction sites [34]. The 6.3 kb PacI cassette from pGOAL19 [34] (*hyg*, *sacB*, *lacZ*) was cloned into the sole PacI site. Vectors were verified by restriction digest and sequencing. 5 μg of UV pre-treated plasmid DNA was electroporated into *M*. *tuberculosis* [35] and single cross over (SCO) transformants were selected on medium containing kanamycin, hygromycin and X-gal. A complementing vector (pLB1C’) carrying a copy of *lytB1* under the control of the Ag85A promoter in the single copy integrating plasmid pAPA3 [36] was constructed, using primers LB1C’ F (PacI) 5’ CCC TTA ATT AAG ATG GCT GAG GTG TTC GTG G 3’ and LB1C’ Rev (PacI) 5’ CCC TTA ATT AAG TCA TTG CGC GCG AAC CTG 3’. A complementing vector (pLB2C’) carrying a functional copy of *lytB2* expressed from its native promoter was constructed as follows: the *lytB2* gene and 300 bp of upstream region was amplified using PLB2 F 5’ CCC AGT ACT TTC TTG GCT TCG CTG GC ATC 3’ and LB2C’ Rev 5’ CCC TCA GCG AGG TGA GCG GAG CTC 3’ cloned into pSC-A and the gentamicin integrating cassette (Gm-int) from pUC-Gm-Int [3] was introduced as a *Hind*III fragment to make a single copy integrating plasmid. Merodiploid strains were constructed by electroporating the SCO strains with the complementing plasmids and isolating kanamycin/hygromycin/gentamicin resistant transformants. Double cross overs (DCOs) were isolated in the wild-type and merodiploid backgrounds by streaking cells onto plates lacking antibiotics and selected/screened on medium containing sucrose, X-gal and gentamicin where required as previously described [34]. PCR screening to determine which allele (wild-type or deletion, LB2 Δ Δ’int) was present in the chromosomal location was carried out using gene-specific screening primers. The genotype of selected strains was confirmed by Southern analysis using the AlkPhos Direct system (GE Healthcare) according to the manufacturer’s instructions (S2 Fig).

### Hybrid switching vectors

The 300 bp upstream promoter region of LytB2 was amplified using PLB2 F (ScaI) 5’ CCC AGT ACT TTC TTG GCT TCG CTG GCA TC 3’; PLB2 Rev (ScaI, BglII, NdeI) 5’ CCC CAT ATG GGG GGG GGA GAT CTA GTA CTG GCA TTC AGG GTA CTT TGG G 3’ and cloned into pSM128 via the ScaI site. The same region was cloned into pSC-A (via TA cloning), to make pPLB2. The 3.75 kbp hyg-int cassette, from pUC-Hyg-int [3], was cloned into pPLB2 as a HindIII fragment to generate pPLB2_H-int. LytB fusions were made as follows: primers were designed to amplify the required regions separately and products obtained from a primary PCR. The products were then cleaned and diluted and added to a secondary PCR at equal volumes. The secondary PCR contained the N-terminal forward primer (BglII) and the C-terminal reverse primer (NdeI) for the required fusion, along with a set of bridging primers- which were homologous to the two fragments and designed to join the two pieces together. The products of the secondary PCR were checked by gel electrophoresis, and again cleaned and diluted. A tertiary PCR was then carried out to obtain the final fusion product using the N-terminal forward primer and the C-terminal reverse primer (primers given in S1 Table). The final product was cloned into pPLB2_H-int via BgIII/NdeI and sequence verified. The *M*. *smegmatis* switching vector (pMS_LB) was constructed by amplifying the *M*. *smegmatis ispH* and cloning into pPLB2_H-int via BglII/NdeI (Msm_LB F (BglII) 5’ CCC AGA TCT GTG CAG TTC CCA TGG GCA A 3’, Msm_LB Rev (NdeI) 5’ CCC CAT ATG TCA ACC GCG CGG CGG GCG G 3’. A second LytB1 complementing vector, with the LytB1 gene under the control of the LytB2 promoter (pPLB2_LB1) was made using LB1C2’ F (BglII) 5’ CCC AGA TCT ATG GCT GAG GTG TTC GTG G 3’ and LB1C2’ Rev (NdeI) 5’ CCC CAT ATG GTC ATT GCG CGC GAA CCT G 3’ and cloning into pPLB2_H-int via BglII/NdeI.

Site directed mutagenesis was carried out on pLB2C’, changing LytB2 bases Gly-34>Ser, Asp-41>Glu, His-62>Lys + Glu-63>Gln, Arg-68>Thr, Thr-125>Ala, and Val-152>Thr; and on pPLB2_LB1 to introduce Ala-121>Thr, A-125>Asn, A-128>Arg and Ala-132>Arg simultaneously using primer sets described in S2 Table. Mutations were confirmed by sequencing.

### Gene switching

Gene switching was carried out as previously described [37,38]. Strains carrying integrated plasmids were electroporated with an integrating plasmid carrying an alternative selection marker and transformants isolated by selecting for the incoming plasmid. Switching was confirmed by patch testing for the loss of the appropriate resistance and PCR or Southern blotting where appropriate. The presence of the *M*. *smegmatis* gene in switching experiments was confirmed by PCR amplification and sequencing.

## Results

### LytB1 is not functionally equivalent to LytB2 in M. tuberculosis

We previously demonstrated that several genes encoding enzymes of the DOXP pathway are essential for the viability of *M*. *tuberculosis* in culture [2–4,13]. *M*. *tuberculosis* has two annotated homologs of LytB (Fig 1) [9,39], although *lytB1* has been identified as producing a enzymatically functional LytB [27], predictions suggest that only *lytB2* is required for *in vitro* growth and that *lytB1* can be inactivated [40,41]. We wanted to determine if *lytB2* was essential for growth *in vitro*.

We attempted to construct an in-frame, unmarked deletion of *lytB2* in a wild-type background, and in a merodiploid background (in which a functional copy of the gene was provided on an integrating plasmid). We were unable to isolate a chromosomal deletion in the wild-type background (0/40 DCOs tested) suggesting the gene is essential. We confirmed this using the merodiploid strain; 18/40 DCO strains tested had chromosomal deletions of *lytB2* (p = 3 x10^-5^ using Fisher’s exact test). The expected genotypes of selected strains were confirmed by Southern analysis.

We next confirmed essentiality by attempting to remove the integrated functional copy of *lytB2* by gene switching. We transformed the del-int strain (chromosomal deletion and functional integrated copy (genotype *lytB2Δ* [*lytB2*, *Gm*, *L5-int*,]) with an “empty” vector (pUC-Hyg-Int) and selected for replacement of the resident integrated vector with the incoming vector (this occurs at high frequency in *M*. *tuberculosis*) [37,38]. We confirmed that the complementing vector could not be removed when the chromosomal gene copy had been deleted. Replacement of the integrated vector in a strain with a functional copy of *lytB2* held on a plasmid with a different antibiotic marker occurred at a high frequency of *~*10^7^ per μg DNA. These data confirm that viability is dependent on the presence of the complementation vector and that *lytB2* is required for growth in culture in *M*. *tuberculosis*.

### Lack of complementation is not due to lack of expression of LytB1

Our data suggested that LytB1 was unable to complement the loss of LytB2, since we were unable to make a deletion strain of the latter, and deletion strains of the former are viable [40,41]. We considered the possibility that *lytB1* is not expressed, or is expressed at a low level and this might account for its inability to compensate for the loss of *lytB2*. Therefore, we constructed an integrating vector in which *lytB1* was under the control of the *lytB2* promoter to ensure expression (pPLB2_LB1). We first confirmed this region had promoter activity using a LacZ reporter gene [42,43]–the *lytB2* upstream region of 300 bp was active as a promoter in *M*. *tuberculosis*, with activity of 161 ± 52 Miller units (compared with 24 ± 6 MU for the negative control containing LacZ with no promoter). The plasmid carrying P_lytB2_-*lytB1* was unable to complement for the *lytB2* deletion, as we were unable to obtain viable transformants after gene switching. Thus we confirmed that LytB1 is not genetically equivalent to LytB2 in *M*. *tuberculosis*.

### M. smegmatis LytB can functionally complement for M. tuberculosis LytB2

**Fig 2:**
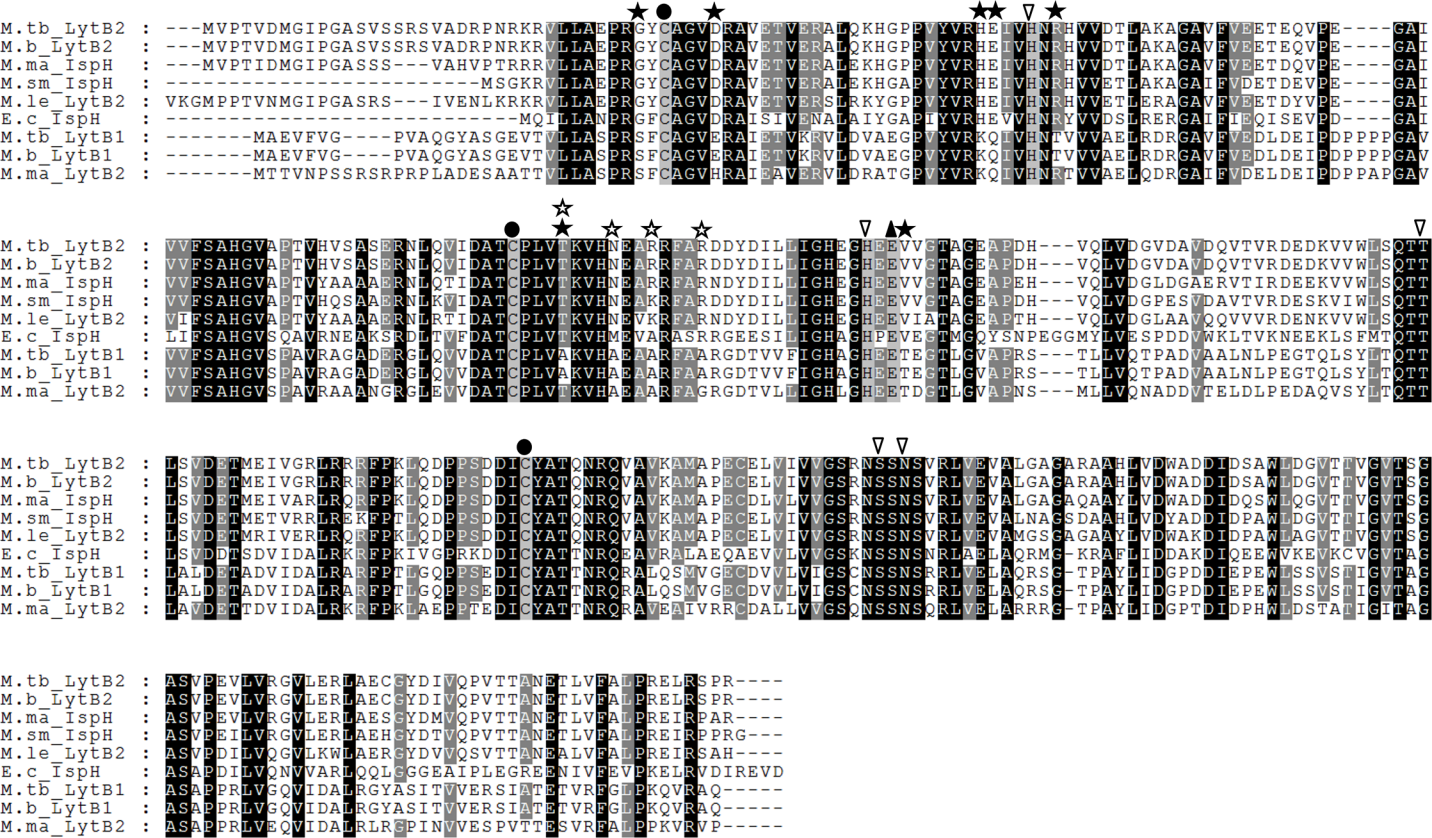
Alignment of LytB homologs. Sequences from *M*. *tuberculosis* (M.tb), *M*. *bovis* (M.b), *M*. *marinum* (M.ma), *M*. *smegmatis* (M.sm), *M*. *leprae* (M.le), *and E*. *coli* (E.c) LytB/IspH homologues were aligned using ClustalW. *M*. *tuberculosis* LytB2, *M*. *bovis* LytB2, *M*. *marinum* IspH, *M*. *smegmatis* IspH and *M*. *leprae* LytB2 form one distinct group, with *M*. *tuberculosis* LytB1 showing a greater sequence identity with *E*. *coli* IspH, *M*. *bovis* LytB1 and *M*. *marinum* LytB2. Black circles indicate equivalents of *E*. *coli* Cys-12, Cys-96, Cys-197, required for Fe-S cluster formation [14–17,24]; the black triangle indicates the critical catalytic residue Glu-126 [26]; white inverted triangles indicate His-41, His-124, Thr-167, Ser-225, Asn-227 required for substrate binding to the active site [17,20,22,24]; black stars indicate bases changed in the LytB2 mutant alleles; white stars indicate the bases changed in the LytB1 mutant allele.

We assessed the distribution of LytB alleles across the mycobacteria. Interestingly, both *M*. *smegmatis* and *M*. *leprae* possess a single LytB homolog, whereas *M*. *tuberculosis*, *M*. *bovis* and *M*. *marinum* all have two isoforms. Alignment of LytB sequences reveals that there are two distinct groups, those that are more similar to *M*. *tuberculosis* LytB2, which include *M*. *bovis* (Mb1140), *M*. *marinum* (MMAR_4355), *M*. *smegmatis* (MSMEG_5224), and *M*. *leprae* (ML1938c); and the second group with more similarity to *M*. *tuberculosis* LytB1 including: *E*. *coli* (IspH), *M*. *bovis* (Mb3414c) and *M*. *marinum* (MMAR_0277) (Fig 2).

It was interesting that the non-pathogenic species *M*. *smegmatis* had only one homologue of *lytB*, despite its genome being almost twice the size of that of *M*. *tuberculosis* [44,45]. We wanted to determine if the *M*. *smegmatis* gene was a true homolog of LytB2 and if it could function in *M*. *tuberculosis*.

We constructed a complementing vector carrying *M*. *smegmatis lytB* (MSMEG_5224) under the control of the *M*. *tuberculosis lytB2* promoter and attempted to switch this into our chromosomal deletion strain. We were able to isolate recombinant clones with hygromycin resistance (and gentamicin sensitivity) indicating that the incoming vector had replaced the resident vector. We confirmed this by PCR and sequencing. Thus the *M*. *smegmatis* allele can complement for the *M*. *tuberculosis* allele and is a true homolog.

### Understanding the critical residues required for a functional LytB

We wanted to explore the differences between the LytB homologs in *M*. *tuberculosis*. In order to delineate the functional regions of the two proteins, we constructed translational fusions and tested these for function in *M*. *tuberculosis* by gene switching (Table 1). We constructed fusions with a varying proportion of LytB1/LytB2 including the N-terminal region of either LytB1 or LytB2. We assessed functionality by gene switching into the del-int strain; if gene switching occurred then we considered the allele to be functional. The only fusion that was functional was where ~90% of the N-terminal of LytB2 was fused to the last 10% of the LytB1 protein. Fusions with 50–75% of LytB2 were not functional; no fusions containing the N-terminal of LytB1 were functional. This suggests that the lack of function of LytB1 is not limited to a small region of the protein or a few amino acid changes. Alternatively, it could be that the fusion proteins lack function due to secondary structure perturbations. In order to address this point we generated more precise mutations in each allele.

**Table 1:** Identification of functional alleles of LytB in *M*. *tuberculosis*.

Plasmid	Allele	Complementation
pPLB2_LB1	LytB1 under control of P_lytB2_	No
pPLB2_LB2	LytB2 under control of P_lytB2_	Yes
pMS_LB	*M*. *smegmatis* LytB	Yes
p50 (1)	50:50 B1:B2 fusion	No
p50 (2)	50:50 B2:B1 fusion	No
p75 (1)	75:25 B1:B2 fusion	No
p75 (2)	75:25 B2:B1 fusion	No
P90 (1)	90:10 B1:B2 fusion	No
p90 (2)	90:10 B2:B1 fusion	Yes
pFeS.1	LytB2 G35S	Yes
pFeS.2	LytB2 D41E	Yes
pFeS.3	LytB2 H62K E63Q	Yes
pFeS.4	LytB2 R68T	Yes
pFeS.5	LytB2 T125A	Yes
pFeS.6	LytB2 V152T	Yes
pFeS.7	LytB1 A121T, A125N, A128R, A132R	No

Switching vectors containing the indicated alleles were transformed into the *M*. *tuberculosis* LytB2 del-int strain carrying an integrated versions of *lytB2* associated with the gentamicin resistance gene (genotype *lytB2Δ* [*lytB2*, *Gm*, *L5-int*,]). pFeS.1-6 LytB2 base numbering, pFeS.7 LytB1. Transformants were selected on hygromycin carried by the incoming vector; gene switching was confirmed by checking for hygromycin resistance and gentamicin sensitivity.

The crystal structure for *A*. *aeolicus*, *E*. *coli*, and *P*. *falciparum* LytB enzymes have been resolved [14,21,25] and key residues have been identified for *E*. *coli*, *A*. *aeolicus*, and *Arabidopsis thalina* alleles [14,21,23–25]. We mapped the conserved residues proposed to be required Fe-S cluster formation [14–17,24,25], for the delivery of H^+^ to the active site [25,26]), and for ligand docking of the substrate into the central cavity [17,20,22,24,25], onto LytB1 and LytB2. All the conserved residues were found in both LytB1 and LytB2 (Fig 2), suggesting that both alleles should be functional. However there were other residues in the same region that were different between the two proteins (Fig 2). For example, the structure of LytB suggests at least one Fe-S cluster and the nucleotide polymorphisms between the two proteins could perturb this site. In order to address this question, we mutated individual residues in LytB2. We selected the residues for mutation based on their proximity to conserved or key residues. Each mutant allele was tested for functionality by gene switching. We also constructed a mutant LytB1 allele in which four alanine residues located in an otherwise highly conserved area were changed to the corresponding amino acid of LytB2, since we postulated that these may have abrogated activity, since alanine lacks a reactive side chain and so rarely contributes to protein function.

Plasmids were transformed into the del-int strain by gene switching and recombinants obtained. All plasmids carrying LytB2 mutant alleles successfully switched indicating that these alleles are functional in *M*. *tuberculosis*. Thus, these residues do not play a key role in LytB function in *M*. *tuberculosis*. In contrast the LytB1 mutant allele did not lead to viable transformants following gene switching. Thus we were unable to reinstate its activity.

## Discussion

The MEP/DOXP pathway of isoprenoid biosynthesis is an attractive target in the search for novel therapeutics. Successful targeting of this pathway in other organisms has already been achieved, since the antibiotic fosmidomycin inhibits Dxr. We have demonstrated that the majority of the genes of the pathway are essential and therefore that this is a crucial metabolic pathway in *M*. *tuberculosis* worthy of further study. LytB2 has an [Fe_4_S_4_] cluster in common with another member of the MEP/DOXP pathway GcpE (IspG) (15–22, 25, [46]. Of interest, the four iron ‘cubane’ cluster has been suggested as a possible target for drug design [47]. In this study we confirmed that LytB2 is essential, and that LytB1 cannot genetically complement for its activity, suggesting that it could be a viable drug target. In support of this, detailed structural information is being determined and inhibitors of the *E*. *coli* enzyme have been identified (49–53).

There are differences in the distribution of LytB isoforms in the mycobacteria. In species with two homologs, LytB2 is located independently and expressed mono-cistronically, whereas *lytB1* is expressed in an operon with *dxs2* (also non-functional in *M*. *tuberculosis*); and in *M*. *marinum* with a second copy of *gcpE* (*ispG*). To date there is no known metabolic function for either LytB1 or Dxs2, both appear to result from gene duplication and divergence; *lytB1* has been demonstrated encode a functional HDR when expressed in *E*. *coli* [27], however Dxs2 is considered to be a pseudogene [10]. In *E*. *coli* LytB also has acetylene hydratase activity, converting acetylenes to aldehydes and ketones via anti-Markovnikov/Markovnikov addition, where n(1)-O-enolate intermediates bind to the fourth iron present in the [Fe_4_S_4_] cluster [48]. It is possible that one or both of the *M*. *tuberculosis* enzyme has similar activities, although these have not been tested. It is tempting to speculate that the LytB1, which appears more similar to the *E*. *coli* enzyme might play a similar catalytic role, but this remains to be established.

We were unable to generate a version of LytB1 which could complement for the cellular function of LytB2 either by fusion or mutation. This was surprising, given that the key conserved residues are preserved in this enzyme. In contrast the lack of activity of Dxs2 is clearly attributable to loss of a catalytic residue (10). Mann *et al*., (2012) noted that LytB1 had a reduced activity compared with LytB2, and considered that this was possibly due to the truncated N-terminal of LytB1 [27]. However our data using LytB1:LytB2 fusions suggest that the inability of LytB1 to complement for the loss of LytB2 is contained within the entire protein sequence, most likely due to the protein structure. Further work could elucidate the structural differences between these two proteins. The reduced activity of LytB1 and our data which reveals that *lytb1* cannot compensate for the loss of *lytb2*, might suggest that, although LytB1 does have HDR activity, it is below the critical threshold required for *M*. *tuberculosis* viability. Another possibility is that *lytB1* is subject to gene silencing in *M*. *tuberculosis*, at either the transcriptional or translational levels. However, this seems less likely, as gene silencing has not been described in mycobacteria, and the enzyme was expressed in *E*. *coli* [27].

## Supporting Information

S1 Fig The MEP/DOXP pathway. LytB (IspH) is a 4-hydroxy-3-methylbut-2-enyl diphosphate reductase (HDR), it acts as the terminal step of the MEP/DOXP pathway catalyzing the conversion of (*E*)-4-hydroxy-3-methyl-but-2-enyl pyrophosphate (HMB-PP) into IPP and dimethylallyl pyrophosphate (DMAPP).
(TIF)

S2 Fig Southern analysis of recombinants. DNA was extracted from wild type H37Rv (WT), LytB2 Δ’int strains (lanes 1, 3, 4), and a LytB2 single crossover strain (SCO, lane 2), and subjected to XhoI/HindIII digest. Bands of the expected sizes were obtained for the WT (2.86 kb), and LytB2 deletion (1.85 kb) alleles; the SCO (lane 2) had both bands, and the LytB2Δ’int strains (1, 3, 4) had the deletion band (1.85 kb).
(TIF)

S1 Table Primers used for PCR cloning. (XLSX)

S2 Table Primers used for site-directed mutagenesis. (XLSX)
