# Supplementary figures and images for: LytB1 and LytB2 of Mycobacterium tuberculosis Are Not Genetically Redundant
Source: PLoS One. 2015 Aug 26;10(8):e0135638. doi: 10.1371/journal.pone.0135638 (PMC4550268; doi:10.1371/journal.pone.0135638)

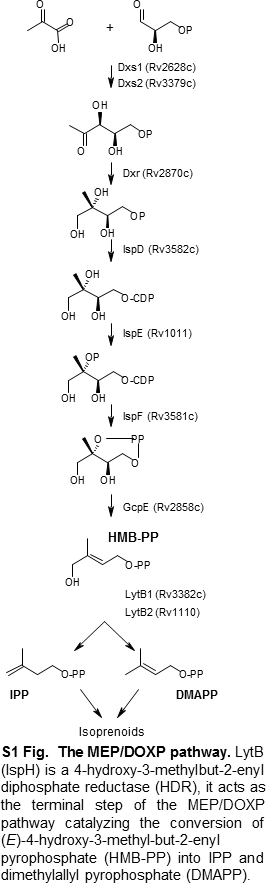

Supplement: S1 Fig — LytB (IspH) is a 4-hydroxy-3-methylbut-2-enyl diphosphate reductase (HDR), it acts as the terminal step of the MEP/DOXP pathway catalyzing the conversion of (E)-4-hydroxy-3-methyl-but-2-enyl pyrophosphate (HMB-PP) into IPP and dimethylallyl pyrophosphate (DMAPP). (TIF) [file pone.0135638.s001.tif]

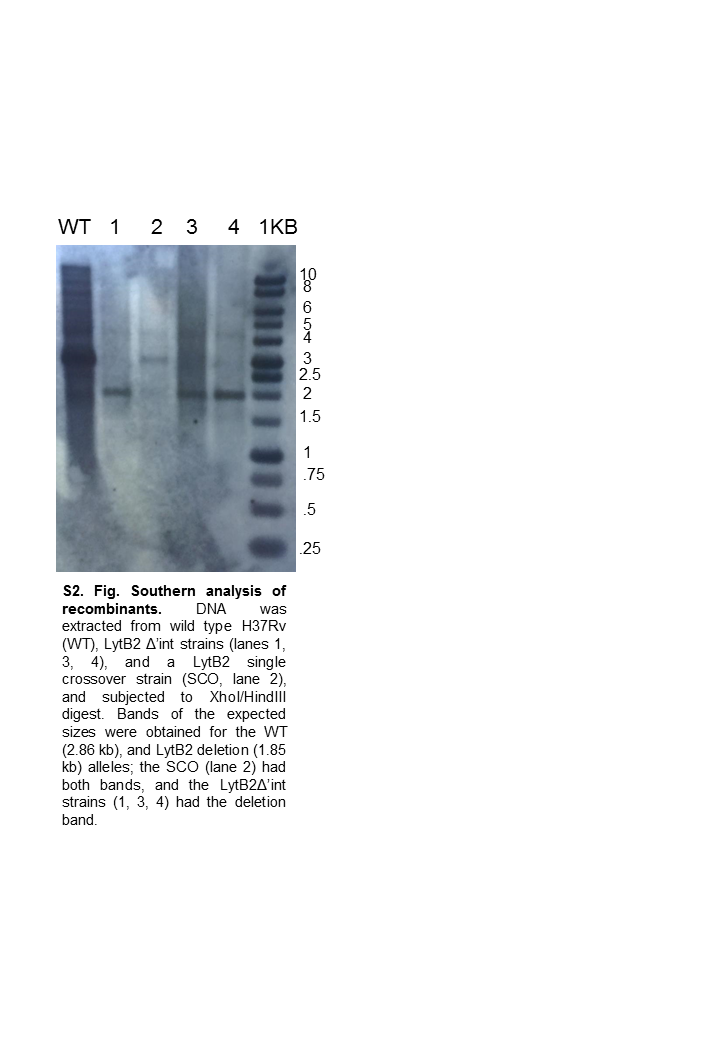

Supplement: S2 Fig — DNA was extracted from wild type H37Rv (WT), LytB2 Δ’int strains (lanes 1, 3, 4), and a LytB2 single crossover strain (SCO, lane 2), and subjected to XhoI/HindIII digest. Bands of the expected sizes were obtained for the WT (2.86 kb), and LytB2 deletion (1.85 kb) alleles; the SCO (lane 2) had both bands, and the LytB2Δ’int strains (1, 3, 4) had the deletion band (1.85 kb). (TIF) [file pone.0135638.s002.tif]
